# Ischemic Stroke and Its Risk Factors in a Registry-Based Large Cross-Sectional Diabetic Cohort in a Country Facing a Diabetes Epidemic

**DOI:** 10.1155/2016/4132589

**Published:** 2016-02-16

**Authors:** Khalid Al-Rubeaan, Fawaz Al-Hussain, Amira M. Youssef, Shazia N. Subhani, Ahmad H. Al-Sharqawi, Heba M. Ibrahim

**Affiliations:** ^1^University Diabetes Center, College of Medicine, King Saud University, P.O. Box 18397, Riyadh 11415, Saudi Arabia; ^2^Department of Medicine, College of Medicine, King Saud University, P.O. Box 2925, Riyadh 11461, Saudi Arabia; ^3^Registry Department, University Diabetes Center, King Saud University, P.O. Box 245, Riyadh 11411, Saudi Arabia; ^4^Department of Biostatistics, Epidemiology and Scientific Computing, King Faisal Specialist Hospital and Research Center, P.O. Box 3345, Riyadh 11211, Saudi Arabia; ^5^Biostatistics Department, University Diabetes Center, King Saud University, P.O. Box 245, Riyadh 11411, Saudi Arabia

## Abstract

The main aim of this study is to determine the prevalence and risk factors of ischemic stroke among diabetic patients registered in the Saudi National Diabetes Registry (SNDR) database. A cross-sectional sample of 62,681 diabetic patients aged ≥25 years was used to calculate ischemic stroke prevalence and its risk factors. Univariate and multivariate logistic regression analyses were used to assess the roles of different risk factors. The prevalence of ischemic stroke was 4.42% and was higher in the older age group with longer diabetes duration. Poor glycemic control and the presence of chronic diabetes complications were associated with a high risk of ischemic stroke. History of smoking and type 2 diabetes were more frequent among stroke patients. Obesity significantly decreased the risk for ischemic stroke. Regression analysis for ischemic stroke risk factors proved that age ≥45 years, male gender, hypertension, coronary artery disease (CAD), diabetes duration ≥10 years, insulin use, and hyperlipidemia were significant independent risk factors for ischemic stroke. We conclude that ischemic stroke is prevalent among diabetic individuals, particularly among those with type 2 diabetes. Good glycemic, hypertension, and hyperlipidemia control, in addition to smoking cessation, are the cornerstones to achieve a significant reduction in ischemic stroke risk.

## 1. Introduction

Disease registries are considered reliable sources for monitoring chronic diseases along with their clinical and economic impacts [1]. Diabetes registries are currently used by many countries to monitor this pandemic and provide accurate scientific data that play a major role in disease management by setting primary and secondary prevention guidelines [2]. The Kingdom of Saudi Arabia, ranked seventh among the top ten countries with a high diabetes prevalence, thereby setting a good example for understanding this disease and its complications [3] through its National Diabetes Registry, which currently contains data of more than one hundred fifty thousand diabetic patients [4].

Diabetes mellitus contributes to approximately one-quarter of all stroke cases [5] and increases the stroke risk by twofold to fivefold. More than 90% of stroke cases are ischemic in nature [6, 7]. Additionally, diabetes worsens the outcome in acute stroke patients, wherein the risk of subsequent stroke increases by twofold and risk of dementia increases by more than threefold among diabetic versus nondiabetic stroke patients [8, 9]. Diabetic patients with stroke are also known for longer hospital stays, worse prognosis, and higher mortality rates [5].

The prevalence of stroke is affected by the presence of various modifiable risk factors, including the degree of glycemic and blood pressure control, smoking, and presence of hyperlipidemia [10, 11], whereas nonmodifiable risk factors, including age, male gender, and diabetes duration, are known to be major contributors to this disease etiology [11]. Racial disparities are also associated with the variable cerebrovascular disease prevalence rate, at 6.9% among American Indians [12], 4.5% among Caucasians [13], and 3.5% among Arabs [14].

There are limited data revealing the extent of ischemic stroke and its risk factors among diabetic patient in the Eastern Mediterranean region; hence using large registry-based data that involve many hospitals would give a realistic assessment of the magnitude of the ischemic stroke and its associated risk factors. The aim of this study was to use a cohort of diabetic patients from the electronic web-based Saudi National Diabetes Registry (SNDR) database to identify the prevalence of ischemic stroke among Saudi diabetic patients and to evaluate its risk factors.

## 2. Materials and Methods

### 2.1. Study Population

The SNDR is an electronic web-based data system that incorporates demographic data and diabetes-related clinical and biochemical parameters based on patients' hospital records. Detailed information about the design and development of the SNDR electronic system has already been described in a previously published paper [4]. A cross-sectional sample of all registered patients from January 1, 2000, to December 31, 2012, was included in this retrospective study; patients younger than 25 years of age were excluded from this study because ischemic stroke is unlikely to occur in this age group.

**Figure 1:**
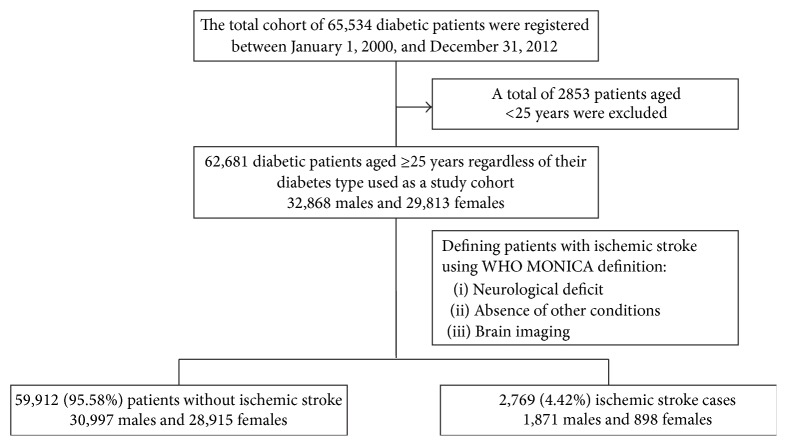
Sample selection flow chart from the total diabetes cohort.

A sample of 62,681 diabetic patients aged ≥25 years composed of 32,868 (52.4%) males and 29,813 (47.6%) females regardless of their diabetes type was included in this study as shown in Figure 1.

Patients data, including: demographic, social, and anthropometric data, and diabetes-related data including type, duration, and the most recent management (i.e., oral hypoglycemic agents, insulin, or both), were collected. Diabetes control parameters: HbA1c, fasting blood sugar (FBS), and random blood sugar (RBS), were also collected. Patients treated for hypertension or with systolic blood pressure (SBP) ≥140 mmHg and/or diastolic blood pressure (DBP) ≥85 mmHg at more than 2 occasions were considered hypertensive, while patients receiving lipid-lowering agents or having total cholesterol ≥5.18 mmol/L or triglycerides ≥1.7 mmol/L were considered hyperlipidemic.

Ischemic stroke was considered based on the clinical presentation and diagnosis supported by brain imaging documented in the patients' files and by following the World Health Organization MONICA Project definition [15], if the clinical symptoms consisted of a rapidly developing neurological deficit that persisted for more than 24 hours or are leading to death in the absence of other conditions that could explain the symptoms. Strokes of nonischemic pathology, that is, intracerebral and subarachnoid hemorrhage, in addition to tumor or venous thrombosis, were excluded based on brain imaging results. All expected risk factors for ischemic stroke were defined using scientific standards, where peripheral vascular disease (PVD) was defined based on either clinical or physical examination documented in patient's medical record, including absent or diminished pulse, abnormal skin color, poor hair growth, and cool skin, or by using ankle brachial index (ABI) measurements, where ABI value of 0.70–0.90 was considered mild occlusion and ABI value of <0.40 was considered as a severe occlusion. Coronary artery disease (CAD) was defined based on the history of hospital admission for either myocardial infarction (MI) or angina, positive electrocardiogram (ECG) testing for prior MI or angina, and positive history of coronary artery bypass grafting or percutaneous transluminal coronary angioplasty (PTCA). Nonproliferative diabetic retinopathy (NPDR) and proliferative diabetic retinopathy (PDR) were defined according to the clinical diagnosis based on the grading of the worst eye. Nephropathy was defined based on the albumin excretion as microalbuminuria when albumin is between 30 and 299 *μ*g/mg creatinine and macroalbuminuria when albumin excretion is ≥300 *μ*g/mg creatinine. End stage renal disease (ESRD) was diagnosed if glomerular filtration rate (GFR) is <30 mL/min per 1.73 m^2^ body surface area. Neuropathy, mainly diabetic polyneuropathy, was considered if the patient presented with lower limb numbness or pain or with an absence of vibration and temperature sensation or a history of foot ulcers and amputation.

SNDR is one of the strategic research projects of Saudi Arabia that was approved and funded by King Abdulaziz City for Science and Technology (KACST) and that can be accessed at http://www.diabetes.org.sa/. However, this database is available for authorized users only. Consent was not obtained for the data used in this publication because this study did not compromise anonymity or confidentiality or breach of local data protection laws.

### 2.2. Statistical Analysis

This study was designed and reported in accordance with Strengthening the Reporting of Observational Studies in Epidemiology (STROBE) guidelines. All data were submitted to the centralized database via the SNDR web application and were analyzed using SPSS version 20.0 (IBM Corp., New York, United States). Descriptive analyses and frequency tables were performed using this program for all variables. The chi-square test (*χ* ^2^) was used for categorical variables such as gender and smoking status, and the *t*-test was used for continuous variables such as age, duration of diabetes, and body measurements, including height, weight, body mass index (BMI), and HbA1c. A *P* value of 0.05 or less was considered significant. Odds ratios (with 95% confidence intervals) were used for assessing ischemic stroke risk factors using the results of univariate analysis, while age- and gender-adjusted model and multivariate logistic regression analysis were used to control for any potential confounders. Imputation was used to estimate missing values from known values using regression model.

## 3. Results

A total of 2769 (4.42%) patients, including 1871 (67.57%) males and 898 (32.43%) females, were diagnosed with ischemic stroke.  Table 1 demonstrates that cases with stroke when compared with diabetic patients without stroke were significantly older at 67.77 (±11.08) years and had lower BMI at 29.28 (±5.97) kg/m^2^ with longer diabetes duration at 17.01 (±8.53) years. The mean HbA1c, FBS, and RBS levels were significantly higher in those with stroke, at 8.89 ± 2.23%, 10.27 ± 4.47 mmol/L, and 13.10 ± 5.65 mmol/L, respectively, compared to patients without stroke at 8.82 ± 2.38%, 9.94 ± 4.27 mmol/L, and 12.64 ± 5.39 mmol/L, respectively. The majority of patients with stroke had type 2 diabetes, whereas only 10.04% were smokers.

Diabetes microvascular complications, namely, neuropathy, retinopathy, and nephropathy, were more frequent among stroked cases at 32.14%, 26.69%, and 19.54%, respectively, while the frequency of macrovascular complications among stroked cases was 26.65% for CAD and 0.98% for PVD. Majority of stroke cases (85.01%) were suffering from hypertension, while 49.01% were having hyperlipidemia and this was significantly higher than nonstroke patients at 45.14% and 35.63%, respectively. More than 65% of patients with stroke used oral hypoglycemic agents; however, the frequency of insulin use was significantly higher among those with stroke (46.98%) compared to those without stroke (36.23%), as shown in Table 2.

**Figure 2:**
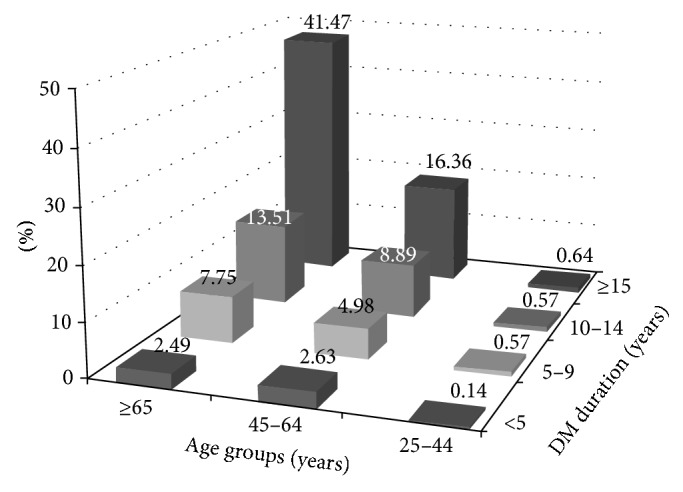
Distribution percentage (%) of stroke patients by age and diabetes duration. The total number of stroke cases, regardless of gender or diabetes type, is 2,769 patients aged ≥25 years.

Figure 2 demonstrates that the percentage of patients with stroke increased exponentially with increasing age and duration being the highest in the age group ≥65 years with duration of diabetes of ≥15 years at 41.47% and the lowest among age group 25 to 44 years with duration less than 5 years at 0.14%. Additional information regarding the effect of gender on the age-specific prevalence of stroke is shown in Table 3, which demonstrates a significantly higher prevalence of stroke among males in all age groups, except for the age group 25–34 years.

Univariate analysis demonstrated that age ≥45 years and male gender indicated significantly higher risk for ischemic stroke. Additionally, age ≥45 years was the strongest risk factor among all analyzed risk factors, with OR (95% CI) at 10.26 (7.46–14.09) as shown in Table 4. Moreover, age ≥45 remained independent as well as the strongest risk factor in the multivariate analysis. Hypertension was the second most important risk factor, with OR (95% CI) at 6.68 (5.87–7.61), and remained a strong risk factor after adjusting for age and gender and even after being examined by multivariate analysis. Hyperlipidemia was also a significant risk factor in univariate and age- and gender-adjusted analyses, as well as in multivariate analysis, but with a lower OR value as shown in Table 5.

The presence of macrovascular complications in the form of PVD and CAD was significantly important risk factors in univariate and age- and gender-adjusted analyses only. However, only CAD was a significant independent risk factor in multivariate analysis. In the univariate analysis of microvascular complications, nephropathy, retinopathy, and neuropathy showed significantly increased risks for ischemic stroke, but when adjusting for age and gender, peripheral neuropathy was the only complication that did not show a significantly increased risk. In contrast, none of the microvascular complications demonstrated independent significant association with ischemic stroke in the multivariate-adjusted model. Diabetes duration ≥10 years was found to be a significant risk factor in univariate and age- and gender-adjusted analyses and remained important independent risk factor in the multivariate regression model. Smoking was a significant risk factor for ischemic stroke in univariate but not in multivariate analysis. Insulin use was also a significant risk factor in all the three regression models, whereas poor glycemic control was not a significant risk factor for ischemic stroke. Obesity, but not overweight status, significantly reduced the risk for ischemic stroke.

Since many other studies have investigated the role of age, gender, diabetes duration, smoking, and obesity as confounders for the association of different risk factors with ischemic stroke, we elected to adjust for these risk factors, where the results show that diabetic nephropathy and retinopathy are insignificant independent risk factors as shown in Supplementary Table 1 (in Supplementary Material available online at http://dx.doi.org/10.1155/2016/4132589), which was not the case when all the risk factors were adjusted in the multivariate model. This analysis emphasized the independent role of all microvascular complications except neuropathy as risk factors for ischemic stroke.

## 4. Discussion

Similar to the findings in Caucasians, as shown by the London cohort of the WHO multinational study of vascular disease in diabetics [13], our cohort had an ischemic stroke prevalence rate of 4.42%, which was higher than what had been reported from the United Arab Emirates (3.5%), which could be a reflection of the lower mean age and/or inclusion of macrovascular stroke cases only [14], but was lower than that of American Indians, who had a reported a prevalence rate of 6.9% [12].

In our cohort, patients with ischemic stroke were significantly older and had longer diabetes duration, where almost 80% of the total stroke cases were older than 45 years with a duration of ≥10 years. This finding is consistent with previous findings that approximately three-quarters of all stroke cases occur in older individuals [16]. In an age-adjusted study of pooled data from 13 prevalence studies, stroke was 41% more prevalent in men than in women [17], which is similar to what we observed and also similar to a more recent study on patients of German ethnicity [18]. The cause of higher stroke incidence among men could be related to genetic factors or could be due to the positive effect of estrogen on cerebral circulation [19]. Another explanation may be the higher prevalence of hypertension, ischemic heart disease, PVD, and smoking, which are also known to be associated with large vessel diseases [20].

Type 2 diabetic patients were the most frequent among ischemic stroke cases and were four times more likely to develop stroke than type 1 diabetic patients. This finding was consistent with the results of other studies that have found type 2 diabetes to be an important risk factor for stroke [21–23].

We studied the effect of a positive family history of diabetes on stroke prevalence and found that patients with stroke have a significantly lower frequency of positive family history when compared to patients without stroke. Since this observation has not been reported previously and because we could not find a clear explanation for this finding, the relationship between the protective effect of a positive family history of diabetes and ischemic stroke requires further investigation.

Old age has been previously reported to be an important risk factor for ischemic stroke [10, 11], and the current study reported age ≥45 years to be the strongest significant risk factor, which is consistent with the findings of the Northern Kentucky Stroke Study in white Americans [24]. However, patients between 45 and 65 years of age had a higher prevalence of stroke in our study than patients aged ≥65 years. This finding is consistent with other studies that showed a higher stroke prevalence among younger populations than among patients aged >55 years [25–27] and a stronger disposition to stroke among diabetic patients compared with nondiabetic patients who have a higher prevalence of hypertension and tend to suffer from stroke at a younger age [25].

Hypertension was an independent risk factor for ischemic stroke in our study, similar to many other studies [28, 29]. Although this issue is currently under debate [30], recent studies have demonstrated the possibility of adverse effects of antihypertensive drugs on glucose and lipid control that could indirectly be behind this risk [31]. Similar to the DAI study and a Taiwanese study [28, 32], we demonstrated that hyperlipidemia is an independent risk factor for ischemic stroke. Although the United Kingdom prospective diabetes study (UKPDS) reported that dyslipidemia was not significantly associated with stroke [7], all cross-sectional design studies similar to ours have demonstrated this positive association [29].

Macrovascular conditions are important risk factors for ischemic stroke in our study, in which CAD and PVD were significantly associated with an increased risk of ischemic stroke. Consistent with the findings of many studies that reported heart disease and cardiac disorders among the common risk factors for stroke in general [33, 34] and among diabetic patients in particular [10, 35], the presence of CAD in the studied cohort was a strong independent risk factor. Although the getABI study had previously reported PVD as a significant independent predictor for ischemic stroke, we found PVD to be a significant risk factor in both univariate and age- and gender-adjusted analyses but not in the multivariate analysis, which could be a reflection of the small number of cases [36].

Diabetic microvascular complications, namely, nephropathy and retinopathy, but not neuropathy, were found to be independent risk factors for ischemic stroke in our study. Although microvascular complications were all significant risk factors in the univariate analysis, similar to many other studies [10, 29, 37], only nephropathy and retinopathy were significant in the age- and gender-adjusted model; which was also observed by Abu El-Asrar et al., reported in Saudi diabetic patients, and Petitti and Bhatt, among Caucasians [38, 39]. The association between retinopathy and ischemic stroke could be explained based on the results of autopsy studies that ischemic stroke in diabetic patients results from small paramedian-penetrating arteries rather than the carotid arteries. Considering this observation and the fact that vascular lesions in the brain are proliferative lesions [40], diabetic retinopathy may predict ischemic stroke because both complications are due to diffuse microvascular complications. Additionally, it was suggested that albuminuria is associated with increase of albumin and fibrinogen transcapillary escape rate, which reflects widespread vascular damage or endothelial dysfunction. Another explanation is that the association between albumin and increased extravascular coagulation leads to the increased release of von Willebrand factor, which contributes to the formation of microthrombi and platelet plugs, followed by areas of nonperfusion [41, 42]. Additionally, diabetic nephropathy and retinopathy significantly increased the risk of ischemic stroke independent of age, gender, diabetes duration, smoking, and obesity. Henceforth, it could be implicated that secondary prevention and early treatment of these complications might reduce the risk of ischemic stroke and its related social and financial ramification.

As previously mentioned, male gender is an independent risk factor in the current study, and this finding is consistent with other studies [11, 14, 28]. A longer diabetes duration was significantly associated with an increased risk of stroke among Taiwanese diabetic patients [28], which is similar to the findings of our study, where the risk increased by approximately 3-fold with a diabetes duration ≥10 years in the multivariate logistic regression model. This observation was similar to the findings of the Northern Manhattan Study, in which the risk of ischemic stroke increased by 3% each year and tripled with a diabetes duration ≥10 years [43].

A history of cigarette smoking was found to be a significant, but not independent, risk factor for ischemic stroke in our study, which was in accordance with Tseng et al.'s [28] observations, which could be related to the small numbers of smokers, especially among women in Saudi society [44].

The BMI was significantly lower among stroke cases in the current analysis; similar findings have been reported among Taiwanese type 2 diabetic patients [28]. However, the association between stroke and obesity remains controversial; the Atherosclerosis Risk in Communities (ARIC) study failed to find a significant association between BMI and risk of stroke [45], along with the Finnmark Study that also found no association between these factors [46]. These findings are more predictive than surprising because many large studies have suggested abdominal obesity, rather than BMI, to be a potent risk factor for stroke [47, 48]. Obesity in our study significantly decreased the risk of stroke, and the same was observed in a Taiwanese study in which BMI was negatively associated with stroke, and a lower BMI was found to be an independent predictor of stroke [28].

In the Hong Kong Diabetes Registry, insulin use was associated with a higher risk of stroke in univariate analysis [35], which was the same finding of our study, in which insulin use was associated with approximately 2-fold increase in the stroke risk in both univariate and age- and gender-adjusted models and remained as an independent risk factor in the multivariate analysis. This finding could partially be explained by insulin's effect on endothelial tissue, where it stimulates the smooth muscle cell proliferation in arterial walls and increases lipid synthesis, leading to lipid lesion formation in arterial tissues. This is in addition to its association with the activation of plasminogen activator inhibitor-1 which is involved in the development of thrombosis [49, 50], as well as the fact that poorly controlled patients are frequent insulin users.

Although many studies have reported a significant positive association between poor glycemic control and increased stroke risk [29, 35], poor glycemic control with a mean HbA1c of 8.89% in our study was associated with a nonsignificant increase in the risk of stroke. This finding could be explained by the fact that our cohort was hospital-based and was subject to better control. This observation is supported by the observation among Caucasians that the risk for stoke was nonsignificant with HbA1c < 9%, whereas it was significant with HbA1c > 9% [51]. Although our study was conducted in a specific ethnic group, the results concerning risk factors can be generalized at an international level.

Our study is limited by its hospital-based retrospective nature that lacks certain specific data which are not retrievable; however, this does not compromise the essential data for case definition and major risk factors. Another limitation of this study is its cross-sectional design, which might have affected the chronological link between stroke events and clinical and metabolic measurements; therefore, this study is not the right setting for determining causality, though it provides enough data to draw significant association between ischemic stroke and the proposed risk factors were associated. In addition, the calculated prevalence might have been affected by unavoidable selection bias since the sample is hospital-based and the primary health care patients were not included. However, as ischemic stroke patients are usually managed at hospital level rather than primary health care centers, this would mean that the vast majority of the cases were included in our registry. Despite these limitations, the strength of our study is derived from a large web-based electronic registry focusing on diabetes and its complications. Additionally, stroke cases analyzed from this large cohort were accurately defined using the WHO MONICA definition that provided an adequate number of cases for better analysis.

## 5. Conclusion

We thereby conclude from this large diabetes registry cohort that the prevalence of ischemic stroke among Saudi type 1 and type 2 diabetic patients was similar to that of other ethnicities and that age ≥45 years and associated diseases such as hypertension are the most important independent risk factors of ischemic stroke. Both micro- and macroangiopathies, in addition to hyperlipidemia, male gender, longer diabetes duration, and insulin use, play significant roles in the etiology of ischemic stroke. Risk reduction can be achieved by good glycemic control, smoking cessation, blood pressure control, and dyslipidemia.

## Supplementary Material

Risk factors of ischemic stroke after adjusting for all the well-established confounders; age and gender and diabetes duration and smoking and obesity to assess the independent association of other risk factors with ischemic stroke.

## Figures and Tables

**Table 1:** Mean (SD) for clinical and biochemical characteristics for the total studied cohort with or without stroke.

Variables	Total studied cohort (62,681)	Patients without stroke (59,912)	Patients with stroke (2,769)	*P* value^*∗*^
	Mean (SD)	Mean (SD)	Mean (SD)	
Age (years)	56.91 ± 13.54	56.59 ± 13.47	67.77 ± 11.08	<0.0001
Weight (kg)	78.86 ± 16.61	78.93 ± 16.62	75.66 ± 15.64	<0.0001
Height (cm)	160.11 ± 9.58	160.11 ± 9.56	160.23 ± 10.31	0.720
BMI (kg/m^2^)	30.63 ± 6.40	30.66 ± 6.40	29.28 ± 5.97	<0.0001
DM duration (years)	13.29 ± 8.10	13.18 ± 8.06	17.01 ± 8.53	<0.0001
HbA1c (%)	8.82 ± 2.37	8.82 ± 2.38	8.89 ± 2.23	0.018
FBS (mmol/L)	9.95 ± 4.28	9.94 ± 4.27	10.27 ± 4.47	<0.0001
RBS (mmol/L)	12.65 ± 5.40	12.64 ± 5.39	13.10 ± 5.65	<0.0001

^*∗*^ *P* value was calculated using nonstroke cohort as a reference.

**Table 2:** Number (%) for clinical and biochemical characteristics for the total studied cohort with or without stroke.

Category	Subcategory	Total studied cohort (62,681)	Patients without stroke (59,912)	Patients with stroke (2,769)	*P* value^*∗*^
		Number (%)	Number (%)	Number (%)	
Age (years)	25–44	11,197 (17.9)	11,135 (18.58)	62 (2.24)	<0.0001
	45–64	31,914 (50.9)	30,996 (51.74)	918 (33.15)	<0.0001
	≥65	19,570 (31.2)	17,781 (29.68)	1,789 (64.61)	<0.0001

Gender	Male	32,868 (52.4)	30,997 (51.74)	1,871 (67.57)	<0.0001
	Female	29,813 (47.6)	28,915 (48.26)	898 (32.43)	<0.0001

Marital Status	Single	2,150 (3.43)	2,113 (3.53)	37 (1.34)	<0.0001
	Married	57,325 (91.46)	54,748 (91.38)	2,577 (93.07)	0.002
	Divorced	641 (1.02)	606 (1.01)	35 (1.26)	0.197
	Widow	2,565 (4.09)	2,445 (4.08)	120 (4.33)	0.512

Family history of DM	Positive	25,187 (40.18)	24,370 (40.68)	817 (29.51)	<0.0001

Smoking	Ever smoked	4,286 (6.84)	3,983 (6.65)	303 (10.94)	<0.0001

BMI (kg/m^2^)	≤25	10,743 (17.14)	10,137 (16.92)	606 (21.89)	<0.0001
	25–29.9	20,855 (33.27)	19,746 (32.96)	1,109 (40.05)	<0.0001
	≥30	31,083 (49.59)	30,029 (50.12)	1,054 (38.06)	<0.0001

Diabetes type	Type 1	2,604 (4.55)	2,568 (98.62)	36 (1.31)	<0.0001
	Type 2	54,669 (95.45)	51,953 (95.03)	2,716 (98.69)	<0.0001

DM duration (years)	<5	8,101 (12.92)	7,951 (13.27)	150 (5.42)	<0.0001
	5–10	18,844 (30.06)	18,341 (30.61)	503 (18.16)	<0.0001
	>10	35,736 (57.01)	33,620 (56.12)	2,116 (76.42)	<0.0001

Neuropathy	Yes	11,153 (17.79)	10,263 (17.13)	890 (32.14)	<0.0001

Retinopathy	Yes	11,262 (17.97)	10,523 (17.56)	739 (26.69)	<0.0001

Nephropathy	Yes	6,252 (9.97)	5,711 (9.53)	541 (19.54)	<0.0001

Vasculopathy	Total	10,384 (16.57)	7,615 (12.71)	2,769 (100)	<0.0001
	CAD	8,020 (12.79)	7,282 (12.15)	738 (26.65)	<0.0001
	PVD	197 (0.31)	170 (0.28)	27 (0.98)	<0.0001

Hypertension	Yes	29,399 (46.90)	27,045 (45.14)	2,354 (85.01)	<0.0001

Hyperlipidemia	Yes	22,701 (36.22)	21,344 (35.63)	1,357 (49.01)	<0.0001

Treatment	Oral agents alone	46,834 (74.72)	45,001 (75.11)	1,833 (66.20)	<0.0001
	Insulin ± oral agents	23,005 (36.70)	21,704 (36.23)	1,301 (46.98)	<0.0001

^*∗*^ *P* value was calculated using nonstroke cohort as a reference.

**Table 3:** Age- and gender-specific prevalence of stroke for the total studied cohort.

Age group	Total		Males		Females		*P* value^*∗*^
	Number	Stroke cases (%)	Number	Stroke cases (%)	Number	Stroke cases (%)	
25–34	3632	5 (0.14)	1616	3 (0.19)	2016	2 (0.10)	0.661
35–44	7565	52 (0.69)	3071	35 (1.14)	4494	17 (0.38)	<0.0001
45–54	17114	305 (1.78)	8154	198 (2.43)	8960	107 (1.19)	<0.0001
55–64	14800	615 (4.16)	7584	344 (4.54)	7216	271 (3.76)	0.017
65–74	13517	1028 (7.61)	7688	634 (8.25)	5829	394 (6.76)	0.001
≥75	6053	764 (12.62)	4755	657 (13.82)	1298	107 (8.24)	<0.0001
Total	62681	2769 (4.42)	32868	1871 (5.69)	29813	898 (3.01)	<0.0001

^*∗*^ *P* value is calculated between the two genders.

**Table 4:** Univariate odds ratio and confidence interval (95% CI) for all cerebrovascular risk factors among studied cohort.

Risk factors	CVD	
	Odds ratio	*P* value
Age ≥ 45 years	10.26 (7.46–14.09)	<0.0001
Hypertension	6.68 (5.87–7.61)	<0.0001
Peripheral vascular disease	3.46 (2.30–5.20)	<0.0001
Coronary heart disease	2.63 (2.41–2.87)	<0.0001
DM duration ≥10 years	2.59 (2.27–2.94)	<0.0001
Nephropathy	2.23 (1.98–2.52)	<0.0001
Male gender	1.92 (1.74–2.12)	<0.0001
Insulin use	1.74 (1.59–1.92)	<0.0001
Smoking	1.72 (1.43–2.08)	<0.0001
Hyperlipidemia	1.71 (1.56–1.88)	<0.0001
Retinopathy	1.71 (1.57–1.86)	<0.0001
Peripheral neuropathy	1.49 (1.23–1.97)	0.005
Poor glycemic control	1.19 (0.95–1.48)	0.127
Overweight	0.94 (0.78–1.13)	0.531
Obesity	0.61 (0.50–0.73)	<0.0001

Risk assessed by univariate logistic regression analysis.

**Table 5:** Age- and gender-adjusted and multivariate-adjusted odds ratio and 95% confidence intervals of risk factors in the studied cohort.

Risk factors	Age- and gender-adjusted			Multivariate-adjusted		
	OR	95% CI	*P* value	OR	95% CI	*P* value
Age ≥ 45 years	—	—	—	4.50	2.87–7.07	<0.0001
Male gender	—	—	—	1.86	1.61–2.15	<0.0001
Hypertension	5.72	5.02–6.52	<0.0001	4.09	3.40–4.91	<0.0001
Peripheral vascular disease	3.07	1.59–5.93	0.001	1.33	0.41–4.34	0.633
Coronary heart disease	2.49	2.22–2.78	<0.0001	1.49	1.27–1.76	<0.0001
DM duration ≥10 years	2.19	1.92–2.50	<0.0001	1.57	1.31–1.88	<0.0001
Nephropathy	1.94	1.72–2.19	<0.0001	1.18	0.99–1.40	0.064
Insulin use	1.86	1.69–2.05	<0.0001	1.44	1.25–1.67	<0.0001
Smoking	1.37	1.13–1.67	0.001	1.23	0.97–1.55	0.086
Hyperlipidemia	1.54	1.41–1.70	<0.0001	1.30	1.13–1.49	<0.0001
Retinopathy	1.35	1.20–1.51	<0.0001	1.01	0.86–1.18	0.897
Peripheral neuropathy	1.10	0.83–1.46	0.515	—	—	—

## Abbreviations

ADA: American Diabetes Association
ABI: Ankle brachial index
ARIC: Atherosclerosis Risk in Communities
BMI: Body mass index
CAD: Coronary artery disease
CI: Confidence interval
DM: Diabetes mellitus
ECG: Electrocardiogram
ESRD: End stage renal disease
FBG: Fasting blood sugar
getABI: German epidemiological trial on ankle brachial index
GFR: Glomerular filtration rate
HbA1c: HemoglobinA1c
KACST: King Abdulaziz City for Science and Technology
MONICA: Monitoring trends and determinants in cardiovascular disease
MI: Myocardial infarction
NPDR: Nonproliferative diabetic retinopathy
OR: Odds ratio
PDR: Proliferative diabetic retinopathy
PTCA: Percutaneous transluminal coronary angioplasty
PVD: Peripheral vascular disease
RBS: Random blood sugar
SD: Standard deviation
SNDR: Saudi National Diabetes Registry
UKPDS: United Kingdom prospective diabetes study
WHO: World Health Organization.
