# Supplementary material for: Ischemic Stroke and Its Risk Factors in a Registry-Based Large Cross-Sectional Diabetic Cohort in a Country Facing a Diabetes Epidemic
Source: J Diabetes Res. 2016 Feb 16;2016:4132589. doi: 10.1155/2016/4132589 (PMC4771899; doi:10.1155/2016/4132589)
Supplement: Supplementary file 1 — Risk factors of ischemic stroke after adjusting for all the well-established confounders; age and gender and diabetes duration and smoking and obesity to assess the independent association of other risk factors with ischemic stroke. [file 4132589.f1.pdf]

Supplementary Table 1: Multivariate – adjusted* odds ratio and 95% confidence intervals of risk factors in the studied cohort:

Risk factors	OR	95% CI	p-value
Hypertension	4.93	3.67-6.61	<0.0001
Peripheral vascular disease	1.36	0.18-10.15	0.762
Coronary heart disease	2.48	1.92-3.20	<0.0001
Nephropathy	1.94	1.52-2.48	<0.0001
Insulin use	1.97	1.57-2.48	<0.0001
Hyperlipidemia	1.61	1.29-2.01	<0.0001
Retinopathy	1.47	1.15-1.87	0.002
Peripheral Neuropathy	0.99	0.58-1.71	0.996

* Adjusted for age , gender , smoking , DM Duration, and obesity
